# Endoplasmic Reticulum Stress Drives VEGF Gene Expression in Monocytic Cells

**DOI:** 10.3390/cimb47100839

**Published:** 2025-10-13

**Authors:** Fatemah Bahman, Taha Nadeem, Abdulrahman Alayyaf, Ashraf Al Madhoun, Fahd Al-Mulla, Sardar Sindhu, Rasheed Ahmad

**Affiliations:** 1 Immunology and Microbiology Department, Dasman Diabetes Institute, Dasman 15462, Kuwait; fatemah.bahman@dasmaninstitute.org; 2 Continental Medical College, University of Health and Sciences, Lahore 53700, Pakistan; tahanadeemk9@gmail.com; 3 College of Medicine, University of Sharjah, Sharjah 27272, United Arab Emirates; itsmealayyaf1@gmail.com; 4 Animal and Imaging Core Facilities, Dasman Diabetes Institute, Dasman 15462, Kuwait; ashraf.madhoun@dasmaninstitute.org; 5 Translational Research Department, Dasman Diabetes Institute, Dasman 15462, Kuwait; fahd.almulla@dasmaninstitute.org

**Keywords:** metabolic stress, obesity, inflammation, ROS, VEGF

## Abstract

Obesity is characterized by chronic low-grade inflammation and oxidative stress, conditions that disrupt metabolic homeostasis and promote vascular endothelial growth factor (VEGF) expression. While hypoxia and fatty acid-induced oxidative stress are known regulators of VEGF, the contribution of endoplasmic reticulum (ER) stress in monocytic cells remains unclear. In this study, we investigated the interplay between ER stress and metabolic stress in regulating VEGF expression using THP-1 monocytic cells. Metabolic stress was induced by palmitic acid (PA) and ER stress by thapsigargin (TG). Co-treatment with PA and TG significantly increased VEGF mRNA and protein levels compared to PA alone. This effect was accompanied by enhanced reactive oxygen species (ROS) production and upregulation of ER stress markers, including CHOP, ATF6, and IRE1. Pretreatment with the antioxidant curcumin markedly reduced VEGF expression and ROS levels, indicating a ROS-dependent mechanism. Additionally, PA+TG co-treatment elevated transcripts of antioxidant defense genes such as SOD2 and NRF2, suggesting a compensatory cellular response to oxidative stress. These findings demonstrate that ER stress amplifies VEGF induction in monocytic cells under lipotoxic conditions through ROS-mediated pathways, highlighting a potential mechanism linking metabolic stress, inflammation, and angiogenesis in obesity-related disorders.

## 1. Introduction

Obesity is a chronic condition marked by the abnormal accumulation of adipose tissue, which interferes with homeostatic regulatory mechanisms and leads to metabolic complications [1]. A key characteristic of this clinical condition is the development of chronic low-grade inflammation, referred to as meta-inflammation in adipose tissue. This results from immune cell infiltration, irregular adipokine production, and impaired metabolic signaling [2,3]. Adipocytes in individuals with obesity exhibit increased secretion of proinflammatory cytokines, including tumor necrosis factor-alpha (TNF-α), interleukin-6 (IL-6), and monocyte chemoattractant protein-1 (MCP-1), which facilitate the recruitment of macrophages and other immune cells that further enhance inflammatory signaling via the activation of transcription factors such as nuclear factor-kappa B (NF-κB) [4,5]. Obesity alters the redox equilibrium within adipose tissue, resulting in excessive production of reactive oxygen species (ROS), primarily originating from mitochondria, NADPH oxidase (NOX) complexes, and endoplasmic reticulum stress responses [6,7].

The ROS not only induces oxidative impairment to various cellular components but also functions as secondary signaling molecules that enhance inflammatory cascades, thereby creating a perpetuating cycle of inflammation and oxidative stress [8]. Importantly, ROS production in adipocytes and infiltrating immune cells is tightly regulated by fatty acid composition; saturated fatty acids (SFAs) such as palmitate have been shown to activate toll-like receptor 4 (TLR4) and stimulate ROS generation via the NOX pathway, while unsaturated fatty acids may exert anti-inflammatory and antioxidant effects [9,10,11].

At the interface of inflammation and oxidative stress lies vascular endothelial growth factor (VEGF), a key angiogenic cytokine whose expression is tightly regulated by hypoxia-inducible factor 1α (HIF-1α), a transcription factor stabilized under conditions of hypoxia and oxidative stress. In obese adipose tissue, the rapid expansion of adipocytes and poor vascular remodeling result in local hypoxia, which induces HIF-1α stabilization and drives VEGF expression [12,13,14]. VEGF is known for its role in promoting neovascularization; emerging evidence implicates it as a proinflammatory mediator that enhances vascular permeability and facilitates leukocyte extravasation [15]. VEGF can directly activate endothelial cells through VEGF receptor-2 (VEGFR2), stimulating MAPK and PI3K/Akt signaling cascades that further perpetuate inflammatory responses [16,17]. In addition, free fatty acids (FFAs), which are elevated in obesity, have been shown to modulate VEGF expression through multiple mechanisms. SFAs upregulate VEGF via TLR4-induced NF-κB activation and ROS-mediated HIF-1α stabilization, whereas polyunsaturated fatty acids (PUFAs) may inhibit VEGF expression through anti-inflammatory effects [18]. Thus, fatty acids serve as critical modulators of both angiogenic and oxidative responses in adipose tissue, with differential effects depending on their saturation status. Understanding the molecular mechanisms by which fatty acids influence VEGF and ROS pathways in obesity not only explains the pathophysiology of metabolic inflammation but also highlights potential intervention points to disrupt the progression of obesity-related complications.

LPS and free fatty acids, including palmitic acid (PA) and oleic acid (OA), are increased in the circulation of individuals with obesity and metabolic syndrome. Circulating free fatty acids are elevated in obesity due to increased adipose tissue lipolysis, impaired peripheral uptake/oxidation, elevated dietary intake, and altered lipoprotein metabolism associated with insulin resistance and low-grade inflammation. PA, the most abundant circulating saturated fatty acid, is commonly used to model lipotoxic metabolic stress because it promotes ROS generation, inflammatory signaling, and insulin resistance, whereas OA, the predominant monounsaturated fatty acid, is often included as a comparator due to its distinct (and typically less cytotoxic) effects on metabolic and inflammatory pathways [19,20]. These changes are also associated with hyperlipidemia, chronic low-grade inflammation, and insulin resistance [21,22]. We hypothesized that the interplay between ER and metabolic stresses contributes to the regulation of VEGF expression in monocytic cells. To explore this, we examined the potential synergistic effect of ER and metabolic stress in promoting inflammation under metabolic disease conditions. Our findings demonstrate that ER stress induced by thapsigargin (TG), when combined with metabolic stress triggered by palmitic acid (PA), leads to an enhanced expression of VEGF in THP-1 cells.

## 2. Materialsand Methods

### 2.1. Cell Culture

THP-1 human monocytic cells were purchased from the American Type Culture Collection (ATCC, Manassas, VA, USA), and cells were cultured in RPMI-1640 medium (Gibco, Life Technologies, Grand Island, NY, USA)) containing 10% fetal bovine serum (FBS; Gibco, USA), 2 mM glutamine (Gibco, USA), 1 mM sodium pyruvate, 10 mM HEPES, 50 U/mL penicillin, and 50 µg/mL streptomycin (Gibco, USA). For in vitro experiments, THP-1 cells were plated (1 × 10^6^ cells/mL/well) in triplicate wells with PA (200 µM), TG (1 µM), PA+TG, and vehicle treated with 0.1% bovine serum albumin (BSA) (control), followed by incubation at 37 °C in a humidified incubator (5% CO_2_) for 24 h, unless otherwise stated. Then, cells were collected and lysed in RLT buffer (Qiagen, Valencia, CA, USA) for total RNA extraction (Qiagen, USA) and in RIPA lysis buffer (Cell Signaling Technology Inc., Danvers, MA, USA) for total protein extraction, following the manufacturer’s instructions. The concentrations of palmitic acid (200 µM) and thapsigargin (1 µM) used here are within non-cytotoxic ranges reported for THP-1 cells in metabolic and ER stress studies. Moreover, the induction of stress-response and antioxidant genes (NRF2, SOD2) indicates that cells remained metabolically active. Thapsigargin (TG) is used as a prototypical ER stress inducer because it specifically inhibits sarco/endoplasmic reticulum Ca^2+^-ATPase (SERCA), causing ER Ca^2+^ depletion, accumulation of misfolded proteins, and activation of the unfolded protein response (UPR). TG, therefore, provides a robust and well-characterized method to probe ER stress-dependent signaling in cell models.

In experiments involving ROS scavengers or antioxidants, THP-1 cells dispensed in triplicate wells (1 × 10^6^ cells/mL/well) were pre-incubated for 1 h with curcumin (10 µM; Cas. #458-37-7; Sigma, Saint Louis, MO, USA), followed by stimulation with metabolic and/or ER stressors as described previously. Notably, antioxidant control wells were pretreated with 0.1% BSA and then stimulated in the same way as other cells that were pretreated with antioxidants. After 24 h of incubation, cell supernatants were collected, aliquoted, and stored at −80 °C for measuring VEGF concentrations by ELISA.

### 2.2. Real-Time Quantitative Reverse Transcription

Total RNA was extracted using an RNeasy kit, following the manufacturer’s instructions. RNA was quantified (Epoch™ Spectrophotometer System; BioTek, Shoreline, WA, USA), and 1 µg RNA sample was used to prepare cDNA using TaqMan reagents (high-capacity cDNA reverse transcription kit; Applied Biosystems, Foster City, CA, USA) [23]. For real-time RT-PCR, a 50 ng cDNA sample was amplified using TaqMan^®^ Gene Expression Master Mix (Cat. #4369016; Applied Biosystems, USA) and TaqMan Gene Expression Assay (Cat. #4331182; Applied Biosystems, USA), using target gene-specific products including VEGF (Hs00900055_m1), CHOP (Hs00358796_g1), ATF6 (Hs00232586_m1), IRE1α (Hs00980095_m1), SOD2 (Hs00167309_m1), NRF2 (Hs00202227_m1), and GAPDH (Hs02786624_g1) containing forward and reverse gene-specific primers and a target-specific TaqMan^®^ 50-FAM-labeled and 30-NFQ-labeled MGB probe, using the Fast Real-Time PCR System (Applied Biosystems, USA). Each cycle consisted of denaturation (95 °C, 15 s) and annealing/extension (60 °C, 1 min), following activation of the uracil-DNA glycosylase (UDG) (50 °C, 2 min) and the AmpliTaq Gold enzyme (95 °C, 10 min). Target gene expression relative to that in the control sample was calculated by the comparative 2^−∆∆CT^ method; data were normalized to GAPDH expression and expressed as fold change over the average gene expression in the control sample, which was taken as 1.

### 2.3. Enzyme-Linked Immunosorbent Assays

The ELISA was performed following the manufacturer’s instructions (VEGF-ELISA kit, Cat. # DVEC00; R&D Systems Inc., Minneapolis, MN, USA). Briefly, cell supernatants and standards were added (100 µL/well) to triplicate wells and incubated at room temperature for 2 h. After aspiration/washing 3 times, the detection antibody was added (100 µL/well) and incubated for 2 h. After 3 washes, a working dilution of streptavidin–HRP was added (100 µL/well) and incubated in the dark for 20 min. After 3 washes, substrate was added (100 µL/well) and incubated in the dark for 20 min. Lastly, a stop solution was added (50 µL/well), optical density (O.D.) was read at a wavelength of 450 nm (correction at 540 or 570 nm), and VEGF concentrations in cell supernatants were calculated from the standard curve.

### 2.4. ROS Detection Assay

To measure intracellular ROS, THP-1 cells were stimulated with PA (200 µM) alone or with TG (1 µM), and the control was treated with only the vehicle (0.1% BSA). After incubation at 37 °C in a humidified incubator (5% CO_2_) for 24 h, ROS induction was measured using a ROS assay kit (Cat. #KP-06-003 BQC Kit; BioQueChem Inc., Llanera-Asturias, Spain), based on uptake of the cell-permeant fluorogenic probe 20–70 dichlorofluorescein diacetate (DCFH-DA). Following cell incubation with the labeled probe for 15 min, DCFH-DA is hydrolyzed by cellular esterases into DCFH carboxylate, which is oxidized by intracellular ROS into the fluorescent 20-70 dichlorofluorescein (DCF) product, which is measured by flow cytometry. After the end of incubation, cells in culture media were loaded with the DCFH-DA probe (15 µM), incubated at 37 °C for 30 min, and analyzed (without washing) by flow cytometry. The final DCF product was excited using a 488 nm laser and detected at a wavelength of 535 nm, and intracellular ROS induction was expressed as mean fluorescence intensity (MFI) values.

### 2.5. Statistical Analysis

The data were expressed as mean ± SEM values, and group means were compared using one-way or two-way ANOVA, Dunnett’s/Tukey’s multiple comparison tests as appropriate. Spearman correlation analysis was performed to determine associations between different variables. GraphPad Prism 10.5.0 (GraphPad Software, La Jolla, CA, USA) was used for statistical analysis of the data and for graph preparation. All *p*-values ≤ 0.05 were considered significant, and statistical significance was expressed as * *p* < 0.05, ** *p* < 0.01, *** *p* < 0.001, and **** *p* < 0.0001.

## 3. Results

### 3.1. The ER and Metabolic Stresses Promote the Expression of VEFG

**Figure 1:**
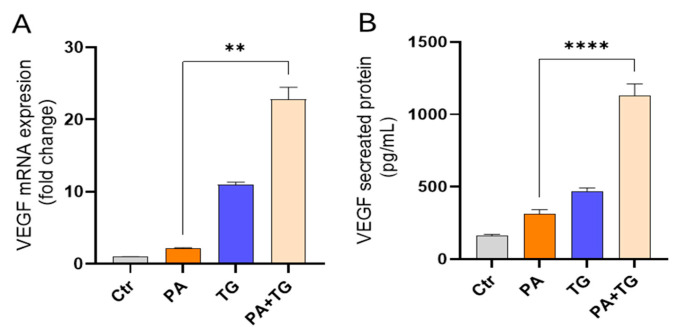
ER stress promotes the metabolic stress-induced VEGF mRNA/protein expression in monocytic cells. THP-1 cells were seeded (1 × 10^6^ cells/mL/well) in triplicate wells and treated with different metabolic stress inducers, including PA (200 μM), in the presence or absence of the ER stressor thapsigargin (TG, 1 μM), while the control was treated only with the vehicle (0.1% BSA), and the cells were incubated for 24 h. Total RNA was extracted from cells to measure VEGF gene expression using qRT-PCR, and cell supernatants were used to detect levels of secreted VEGF protein via ELISA. Similar results were obtained from three independent experiments. Data (expressed as mean ± SEM) were analyzed using one-way ANOVA, Tukey’s multiple comparisons test, and ** *p*-values ≤ 0.001 and **** *p*-values ≤ 0.0001 were considered significant. The representative data show that ER stress (TG treatment) increases: (**A**) VEGF mRNA expression in THP-1 cells that were treated with PA and (**B**) secreted VEGF protein levels in response to treatment with PA.

In obesity, the expansion of adipose tissue is accompanied by persistent low-grade inflammation, stimulated by the circulation of free fatty acids and pro-inflammatory cytokines. Hypoxia develops under conditions of oxidative stress, further impairing tissue function and driving whole-body metabolic dysfunction. However, it is unclear whether ER stress could promote VEGF expression in monocytic cells that are challenged by lipotoxic or metabolic stress. We used human THP-1 monocytic cells in our studies. The THP-1 monocytic cell line is widely used as a representative human monocyte model to study inflammatory and metabolic signaling because it reproduces many phenotypic and transcriptional responses of primary monocytes under stress conditions [24,25,26]. Its homogeneity and reproducibility make it particularly suitable for mechanistic studies of ER and oxidative stress pathways. Our data show upregulated VEGF mRNA expression in THP-1 cells following stimulation with PA+TG (22.84-fold) compared with PA (2.21-fold) (Figure 1A). As expected, VEGF protein levels were also higher in THP-1 cells that were co-stimulated with PA+TG (1131 pg/mL) compared with PA (311 pg/mL) (Figure 1B).

**Figure 2:**
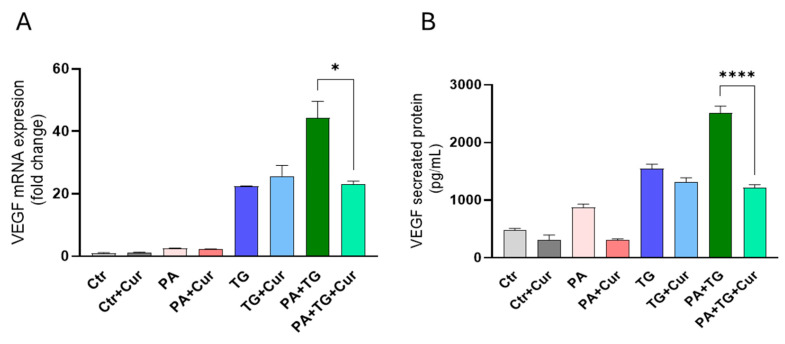
Metabolic stress-induced expression of VEGF mRNA and protein in THP-1 cells. Cells were seeded (1 × 10^6^ cells/mL/well) in triplicate wells and treated with different metabolic stress inducers, including PA (200 μM), in the presence or absence of the ER stressor thapsigargin (TG, 1 μM) and curcumin (curcumin, 1 µM), while the control was treated only with the vehicle (0.1% BSA), and the cells were incubated for 24 h. Total RNA was extracted from cells to measure VEGF gene expression using qRT-PCR, and cell supernatants were used to detect levels of secreted VEGF protein via ELISA. The representative data show that ER stress (TG treatment) increases: (**A**) VEGF mRNA expression in THP-1 cells that were treated with PA, TG, and/or curcumin and (**B**) secreted VEGF protein levels in response to treatments with PA, TG, and/or curcumin. Similar results were obtained from three independent experiments. Data (expressed as mean ± SEM) were analyzed using one-way ANOVA and Tukey’s multiple comparisons test, and *p*-values ≤ 0.05 were considered significant (* *p*-values ≤ 0.01, **** *p*-values ≤ 0.0001).

Then, to test the antioxidant effect of curcumin, we pretreated THP-1 cells with curcumin prior to PA and PA+TG stimulation, as shown in Figure 2, where VEGF gene expression levels for Cu+PA+TG (23.14-fold) were markedly reduced compared to PA+TG-stimulated cells (44.2-fold) (Figure 2A). As expected, VEGF protein levels were lower in THP-1 cells pretreated with curcumin as Cu+PA+TG (1220 pg/mL) compared to those stimulated with PA+TG alone (2515 pg/mL) (Figure 2B).

### 3.2. Metabolic and/or ER Stress(es) Induce(s) Reactive Oxygen Species (ROS)

**Figure 3:**
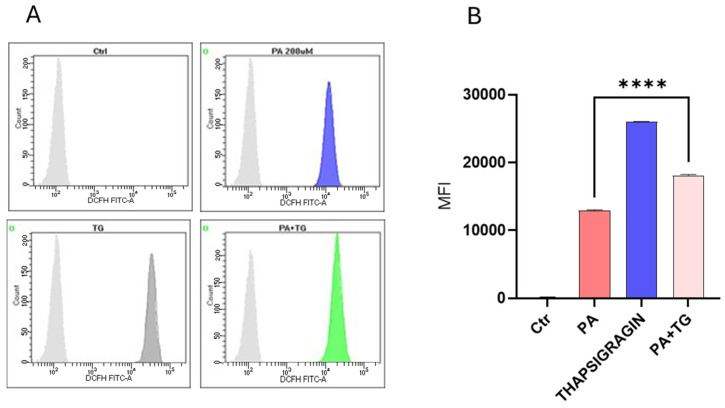
Metabolic and ER stresses induce intracellular reactive oxygen species (ROS). THP-1 cells were plated (1 × 10^6^ cells/mL/well) in triplicate wells and treated with PA (200 μM) in the presence or absence of the ER stressor thapsigargin (TG, 1 μM), while the control was treated only with the vehicle (BSA), and the cells were incubated for 24 h. Intracellular ROS was measured using a DCFH-DA assay and flow cytometry as described in Section 4. Similar results were obtained from three independent experiments. Data (expressed as mean ± SEM) were analyzed using one-way ANOVA and Tukey’s multiple comparisons test, and *p*-values ≤ 0.05 were considered significant (**** *p*-values ≤ 0.0001). (**A**) The representative data show that ER stress (TG treatment) promotes ROS in cells that are metabolically stressed due to PA treatment. (**B**) MFI of PA+TG treatment (MFI: 18,084 ± 152), PA treatment (MFI: 12,934 ± 78), TG treatment (MFI: 25,976 ± 71), and control (MFI: 170 ± 16).

**Figure 4:**
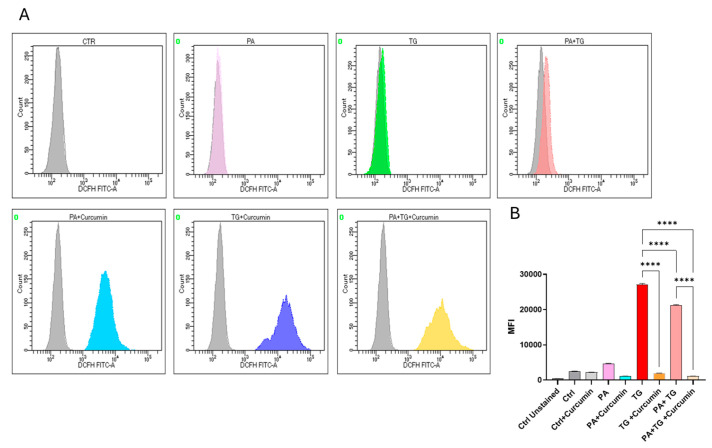
The effect of curcumin on ROS generation by metabolic and ER Stresses: THP-1 cells were treated with PA (200 μM) in the presence or absence of thapsigargin (1 μM) and/or curcumin (1 μM), while the control was treated with only the vehicle (BSA), and the cells were incubated for 24 h. Intracellular ROS was measured using a DCFH-DA assay. (**A**) The representative data show that curcumin reduces ER stress (TG treatment) and metabolic stress (PA treatment). (**B**) MFI of PA (MFI: 4729 ± 48.07), Cur (MFI: 2241 ± 42.76), TG (MFI: 27,136 ± 249), Cur+TG (MFI: 1988 ± 17.25), PA+TG (MFI: 21,211 ± 190), and PA+Cur+TG (MFI: 1120 ± 24.44) treatments. Similar results were obtained from three independent experiments. Data (expressed as mean ± SEM) were analyzed using one-way ANOVA and Tukey’s multiple comparisons test, and *p*-values ≤ 0.05 were considered significant (**** *p*-values ≤ 0.0001).

ER-mediated nutrient sensing is vital for cellular homeostasis, and stress within this organelle may precipitate chronic low-grade inflammation in different tissues and cell populations [27]. Next, we sought to test whether ER and metabolic stresses contribute to ROS induction. ROS levels were measured in THP-1 cells that were treated with PA or with the ER stressor TG. The flow cytometry data show that higher ROS levels were co-induced via ER/metabolic stresses involving PA (Figure 3). In addition, to show that ROS expression represented oxidation-dependent changes and not the effect of the influx/efflux of the DCFH probe due to high lipid load, we treated the cells with the antioxidant curcumin before PA and PA+TG stimulation, and as expected, these data show that pretreatment with curcumin significantly suppressed the expression of ROS (Figure 4).

### 3.3. Lipotoxic Treatments Induce ER Stress in THP-1 Cells

**Figure 5:**
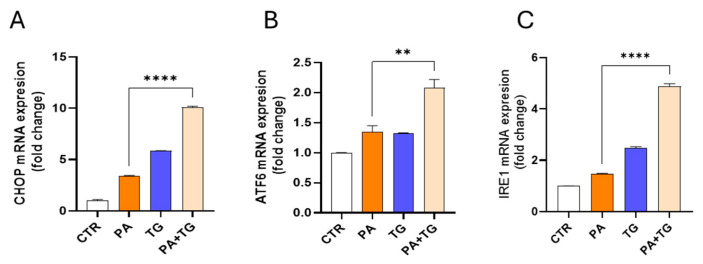
Metabolic stress induces or promotes ER stress. THP-1 cells were treated with PA (200 μM) in the presence or absence of the ER stress inducer thapsigargin (TG, 1 μM), while the control was treated only with the vehicle (0.1% BSA), and the cells were incubated for 24 h. Total RNA was extracted, and the gene expression of ER stress markers including CHOP, ATF6, and IRE1 was determined using qRT-PCR. Similar results were obtained from three independent experiments. Data (expressed as mean SEM) were analyzed using one-way ANOVA and Tukey’s/Dunnett’s multiple comparisons test, and *p*-values 0.05 were considered significant. The representative data show, compared with control, the increased (**A**) CHOP mRNA levels in cells treated with PA, TG, and PA+TG; (**B**) ATF6 mRNA levels in cells treated with PA, TG, and PA+TG; and (**C**) IRE1 mRNA levels in cells treated with PA, TG, and PA+TG. Statistical significance is shown as ** *p* < 0.01, and **** *p* < 0.0001, compared with the respective control (vehicle treatment).

The ER plays a critical role in cellular nutrient sensing, and ER stress may act as a trigger of chronic low-grade inflammation in metabolic syndromes [28]. Next, we tested our hypothesis that metabolic insults induce or elevate ER stress in THP-1 cells. We measured the expression of ER stress sensor transcripts, including CCAAT- enhancer-binding protein (C/EBP) homologous protein (CHOP), activating transcription factor (ATF)-6, and inositol-requiring enzyme (IRE)-1, also known as ER to nucleus signaling (ERN)-1, following cell stimulations with PA alone or in the presence of TG. The results show significant upregulation of CHOP transcripts in response to treatments with PA (3.39-fold), TG (5.84-fold), and PA+TG (10.07-fold) compared with the control, while only PA+TG co-treatment induced higher CHOP expression than that induced by TG alone (*p*-value < 0.0001) (Figure 5A). Similarly, ATF6 expression was induced by treatments with PA (1.35-fold), TG (1.32-fold), and PA+TG (2.08-fold) compared with the control, while PA+TG co-stimulation induced higher ATF6 expression than that induced by TG alone (*p*-value 0.0001) (Figure 5B). IRE1 expression was induced by stimulation with PA (1.46-fold), TG (2.47-fold), and PA+TG (4.87-fold), compared with the control. However, PA+TG co-stimulation induced higher IRE1 expression than that induced by TG alone (*p*-value < 0.0001) (Figure 5C). Taking together, PA+TG stimulation upregulates the multiple pathways of ER stress in THP-1 cells significantly.

### 3.4. Metabolic and ER Stresses Trigger Cellular Antioxidant Defense Mechanisms

**Figure 6:**
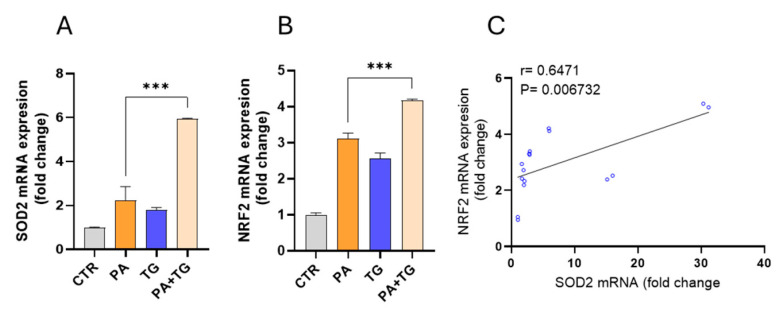
Metabolic and/or ER stress activates antioxidant defense mechanisms. THP-1 cells were treated with PA (200 μM) in the presence or absence of the ER stress inducer thapsigargin (TG, 1 μM), while the control (Ctrl) was treated with only the vehicle (0.1% BSA), and the cells were incubated for 24 h. Total RNA was extracted, and gene expression of SOD2 and NRF2 was determined using qRT-PCR. Similar results were obtained from two independent experiments. Data (expressed as mean ± SEM) were analyzed using one-way ANOVA and Tukey’s or Dunnett’s multiple comparisons test, as appropriate. All *p*-values ≤ 0.05 were considered significant. The representative data show the mRNA expression of (**A**) SOD2 and (**B**) NRF2 in monocytic cells (*p* < 0.001). (**C**) Based on gene expression data, a strong agreement was found between SOD2 and NRF2 (r = 0.6471, *p* = 0.006732). Data expressed as mean ± SEM and were analyzed using one-way ANOVA and Tukey’s or Dunnett’s multiple comparisons test, as appropriate. All *p*-values < 0.05 were considered significant. *** *p* < 0.001.

The induction of oxidative stress is accompanied by the activation of cellular antioxidant defense systems, which are essential for maintaining redox homeostasis. This balance is crucial for preserving cellular viability and proliferation and ensuring suitable function [29]. To this effect, we measured transcript expression of superoxide dismutase (SOD)-2 and nuclear factor erythroid 2-related factor (NRF)-2 in THP-1 cells after metabolic stress challenge with or without TG, and we found increased SOD2 mRNA expression following stimulation with PA+TG (5.94-fold) compared with respective treatments without TG (*p*-value = 0.0004) (Figure 6A). We also found elevated NRF2 mRNA expression in cells that were stimulated with PA+TG (4.16-fold) compared with respective treatments without TG (*p*-value = 0.0006) (Figure 6B). In addition, a strong correlation was found between SOD2 and NRF2 transcript expression in THP-1 cells (r = 0.6471, *p*-value = 0.006732) (Figure 6C). Transcriptional data support that cellular antioxidant defense mechanisms respond to ER and metabolic stresses in THP-1 cells.

## 4. Discussion

The present study demonstrates that ER stress acts as a potent amplifier of VEGF expression in monocytic cells exposed to metabolic stress, and that this effect is mediated, at least in part, by increased ROS production. Our data provide new insight into the crosstalk between lipotoxic and ER stress pathways in the regulation of angiogenic and inflammatory responses relevant to obesity-related disorders.

Previous studies have reported that VEGF expression in adipose tissue is primarily driven by hypoxia via stabilization of HIF-1α, particularly under conditions of rapid adipose expansion and inadequate vascular remodeling [30]. Infiltrating immune cells, including macrophages and monocytes, have also been shown to produce VEGF in response to pro-inflammatory cytokines and free fatty acids [31]. However, the contribution of ER stress to VEGF regulation in immune cells has received comparatively little attention. Our findings extend this body of work by demonstrating that ER stress, induced by thapsigargin, synergizes with palmitic acid to enhance VEGF expression in THP-1 cells beyond that achieved by metabolic stress alone. Although ER stress activation was evaluated at the transcriptional level, prior studies have demonstrated a strong correlation between increased expression of CHOP, ATF6, and IRE1 and the functional activation of the unfolded protein response in THP-1 and other human cells. The observed induction of these genes, together with enhanced ROS generation and VEGF expression, supports activation of ER stress signaling under metabolic challenge. This synergistic effect suggests that ER stress is not merely a byproduct of lipotoxicity but may serve as an active regulator of angiogenic signaling [32].

The observed increase in ROS levels following PA+TG co-treatment is consistent with previous evidence linking ER stress to mitochondrial dysfunction and oxidative stress in metabolic syndrome [33]. ROS are known to function as both damaging agents and signaling intermediates, capable of activating transcription factors such as NF-κB and HIF-1α, which are implicated in VEGF upregulation [34]. VEGF expression is regulated by multiple transcriptional pathways, including HIF-1α, NF-κB, and ATF4, which are redox-sensitive and can be activated downstream of ER and oxidative stress. Although their activation was not directly assessed here, the marked attenuation of VEGF induction by the antioxidant curcumin suggests that ROS generation acts as a major upstream driver. Our results, showing that antioxidant pretreatment with curcumin markedly reduced VEGF expression, support the idea that ROS act as a critical mediator in ER stress-driven angiogenic signaling. This finding aligns with earlier studies in endothelial and cancer cells, where ROS scavenging was found to attenuate VEGF production [35], but it is novel in the context of monocytic cells under combined metabolic and ER stress.

We also observed that PA+TG co-treatment significantly upregulated ER stress markers CHOP, ATF6, and IRE1, indicating activation of multiple branches of the unfolded protein response (UPR). This broad ER stress activation suggests that VEGF induction in our model may be regulated through diverse UPR-linked transcriptional programs, potentially involving CHOP-mediated inflammatory gene expression and ATF6/IRE1-mediated crosstalk with oxidative stress pathways [35]. Prior research has shown that ATF6 can promote angiogenesis through VEGF regulation in endothelial cells, while IRE1 has been implicated in inflammatory signaling [36]. Our findings, therefore, add to the growing evidence that ER stress sensors may integrate metabolic and inflammatory cues to drive VEGF expression.

Interestingly, the co-induction of antioxidant defense genes SOD2 and NRF2 in response to PA+TG suggests that monocytic cells attempt to counteract the increased oxidative load. Similar compensatory NRF2 activation has been reported in adipocytes and hepatocytes under metabolic stress [37], but the extent to which this response can effectively neutralize ROS in chronic inflammatory conditions remains unclear. The concurrent rise in ROS levels and induction of antioxidant genes such as NRF2 and SOD2 likely reflects a compensatory yet insufficient adaptive response. Under chronic metabolic or ER stress, excessive ROS generation can surpass cellular antioxidant capacity, leading to persistent oxidative imbalance despite activation of defense mechanisms. In response to elevated ROS, cells activate conserved sensor–effector pathways: oxidative modification of Keap1 stabilizes and promotes nuclear translocation of NRF2, which binds antioxidant response elements (AREs) to induce genes involved in redox regulation, including SOD2, catalase, and glutathione-synthesizing enzymes. Stress-activated kinases and NF-κB signaling may further modulate this antioxidant program. These mechanisms aim to restore redox homeostasis; however, when ROS production remains high—as under combined lipotoxic and ER stress—the compensatory response becomes inadequate, resulting in sustained oxidative signaling and downstream effects such as VEGF induction. In our model, despite activation of antioxidant defenses, VEGF expression remained elevated, indicating that redox imbalance persisted. In our model, despite the activation of antioxidant defenses, VEGF expression remained elevated, indicating that ROS-mediated signaling persists even in the presence of endogenous protective mechanisms.

Taken together, our results support a model in which ER stress amplifies metabolic stress–induced VEGF expression in monocytic cells through ROS-dependent mechanisms, with potential contributions from multiple UPR pathways. ER stress amplifies metabolic stress responses through several convergent mechanisms. ER Ca^2+^ dysregulation and ER–mitochondrial crosstalk increase mitochondrial ROS production; PERK/ATF4, ATF6, and IRE1 branches of the UPR activate proinflammatory transcriptional programs and can potentiate NF-κB and AP-1 signaling; and UPR signaling can enhance HIF-1α stability under redox-active conditions. These combined effects magnify ROS-dependent and transcriptional drivers of VEGF expression, explaining why ER stress functions as a potent amplifier in the context of palmitate-induced metabolic stress. This mechanism is relevant to obesity, where chronic exposure to saturated fatty acids and inflammatory mediators can sustain ER stress and oxidative stress in immune cells, thereby promoting both angiogenesis and inflammation. By identifying ER stress as a key modulator of VEGF expression in monocytes, our study suggests that targeting ER stress or its downstream ROS production may represent a therapeutic strategy to attenuate pathological angiogenesis and inflammation in obesity-related cardiometabolic diseases. This study was designed as a mechanistic in vitro investigation to delineate the crosstalk between ER and metabolic stress pathways in monocytes. The observed induction of ER stress, ROS, and VEGF is consistent with molecular signatures reported in obese adipose tissue and metabolic syndrome. Nevertheless, further validation using monocytes from obese subjects or animal models will be essential to establish direct clinical relevance.

## 5. Conclusions

In summary, our findings demonstrate that interactions between metabolic and ER stresses drive intracellular ROS generation and markedly enhance VEGF expression in monocytic cells. This suggests that ER–mitochondrial and redox interactions potentiate angiogenic and inflammatory signaling under lipotoxic conditions, contributing to metabolic inflammation. Notably, curcumin pretreatment attenuated VEGF production, highlighting the therapeutic potential of ROS scavengers and antioxidants in conditions characterized by combined metabolic and ER stress. While this study provides mechanistic insight using a cell model, validation in primary monocytes or in vivo models will be important to confirm the relevance of this pathway in obesity-associated tissue remodeling. Additionally, VEGF regulation may involve other transcriptional mediators, such as HIF-1α, NF-κB, and ER-associated factors, which warrant further investigation in future studies.

## Data Availability

The original contributions presented in this study are included in the manuscript. Further inquiries can be directed at the corresponding authors.
